# Subtype distribution of zoonotic pathogen *Cryptosporidium felis* in humans and animals in several countries

**DOI:** 10.1080/22221751.2020.1840312

**Published:** 2020-11-06

**Authors:** Wen Jiang, Dawn M. Roellig, Marianne Lebbad, Jessica Beser, Karin Troell, Yaqiong Guo, Na Li, Lihua Xiao, Yaoyu Feng

**Affiliations:** a School of Resources and Environmental Engineering, East China University of Science and Technology, Shanghai, People’s Republic of China; b Division of Foodborne, Waterborne, and Environmental Diseases, Centers for Disease Control and Prevention, Atlanta, GA, USA; c Public Health Agency of Sweden, Solna, Sweden; d National Veterinary Institute, Uppsala, Sweden; e Center for Emerging and Zoonotic Diseases, College of Veterinary Medicine, South China Agriculture University, Guangzhou, People’s Republic of China

**Keywords:** *Cryptosporidium felis*, subtypes, subtype families, host adaption, geographic distribution

## Abstract

*Cryptosporidium felis* is the major etiologic agent of cryptosporidiosis in felines and has been reported in numerous human cryptosporidiosis cases. Sequence analysis of the 60-kDa glycoprotein (*gp60*) gene has been developed for subtyping *C. felis* recently. In this study, 66 *C. felis* isolates from the United States, Jamaica, Peru, Portugal, Slovakia, Nigeria, Ethiopia, Kenya, China, India and Australia were subtyped using the newly established tool. Forty-four specimens yielded *gp60* sequences, generating 23 subtypes clustered in 4 subtype families (XIXa, XIXc, XIXd and XIXe) with high bootstrap support in a phylogenetic analysis of sequence data. Among them, XIXa showed high genetic diversity at the nucleotide level, with the formation of 18 subtypes from both cats and humans with different geographic distribution. In contrast, all 11 XIXd isolates derived from humans from various countries had identical sequences. Results of this study improve our understanding of the genetic diversity, host specificity and transmission dynamics of *C. felis*.

## Introduction

*Cryptosporidium* spp. are protozoan parasites that can cause significant gastrointestinal disease in humans and animals [1]. Currently, the majority of reported cases of human cryptosporidiosis are caused by several *Cryptosporidium* species, including *C. hominis*, *C. parvum*, *C. meleagridis*, *C. felis*, *C. canis*, *C. cuniculus*, and *C. ubiquitum* [2–5]. Among them, *C. felis* was initially reported in domestic cats from Japan in 1979 and proposed as a new species based on host occurrence and oocyst morphology, which was later supported by genetic characterizations of isolates [6,7]. This species has been reported in numerous human cases subsequently [8], and sometimes in association with the presence of infection in cats in the same household [9,10].

Sequence analysis of the 60-kDa glycoprotein (*gp60*) gene has been widely used in subtyping *C. hominis* and *C. parvum*, with the identification of host-adapted subtype families within both species [11]. Recently, *gp60*-based subtyping tools have been developed for some other *Cryptosporidium* spp. such as *C. meleagridis*, *C. ubiquitum*, *C. viatorum*, *C. ryanae* and *Cryptosporidium* chipmunk genotype I [12–16]. The use of these subtyping tools has significantly improved our understanding of the transmission of *Cryptosporidium* spp., especially the role of anthroponotic and zoonotic infections in cryptosporidiosis epidemiology [2,15].

A *gp60-*based subtyping tool has become available recently for *C. felis* [10]. Thus far, the genetic characterization of *C. felis* has been mostly restricted to isolates acquired in three European countries (the United Kingdom, Sweden and Denmark). In this study, additional *C. felis* isolates mostly from Africa and the Americas were analysed to understand the geographic and host distribution of subtypes within this zoonotic *Cryptosporidium* species.

## Materials and methods

### Specimens

DNA preparations from 66 *C. felis*-positive samples were included in this study, including 58 from humans, 6 from cats, 1 from a calf, and 1 from a rhesus macaque. Human samples were collected in the United States, Jamaica, Peru, Portugal, Ethiopia, Kenya, Nigeria, India, and China; cat samples were from the United States, Peru, Slovakia, and Australia; while the calf sample was from Portugal and rhesus macaque sample from China (Table 1). Genomic DNA samples were stored in –80°C freezer for no more than 20 years prior to PCR analysis in the study. These samples were initially determined to be positive for *C. felis* by PCR and sequence analysis of the small subunit (SSU) rRNA gene as previously described [17].

**Table 1.** *Cryptosporidium felis* isolates used in this study, their subtype identity and copy numbers of major tandem repeats in the *gp60* gene.

Host	Isolate ID	Country	Subtype	No. of 33-bp repeat	No. of 39-bp repeat	No. of GGT
Children	1225	Peru	XIXd-1	1	1	4
	2691	Peru	N/A	–	–	–
	2748	Peru	XIXd-1	1	1	4
	2819	Peru	N/A	–	–	–
	3221	Peru	N/A	–	–	–
	3242	Peru	N/A	–	–	–
	3254	Peru	N/A	–	–	–
	11747	Peru	XIXa-3	2	2	4
	12419	Peru	XIXa-4	2	2	4
	4306	India	N/A	–	–	–
	28340	India	N/A	–	–	–
	28356	India	N/A	–	–	–
	9043	Kenya	XIXe-1	1	1	4
	37146	Kenya	XIXe-2	1	1	4
	37157	Kenya	XIXd-1	1	1	4
	29020	China	N/A	–	–	–
	30382	China	XIXa-14	2	2	4
HIV+ persons	3931	Peru	XIXa-2	2	2	4
	4202	Peru	XIXd-1	1	1	4
	4551	Peru	XIXd-1	1	1	4
	4595	Peru	XIXd-1	1	1	4
	5162	Peru	N/A	–	–	–
	5166	Peru	XIXc-2	2	3	3
	6118	Peru	XIXa-1	2	2	4
	6124	Peru	XIXa-1	2	2	4
	6491	Peru	XIXd-1	1	1	4
	7078	Peru	XIXd-1	1	1	4
	7079	Peru	XIXa-5	2	3	4
	7366	Peru	XIXa-6	2	2	4
	19227	Peru	XIXd-1	1	1	4
	19232	Peru	XIXa-5	2	3	4
	19237	Peru	XIXa-6	2	2	4
	19242	Peru	XIXa-5	2	3	4
	35929	Ethiopia	N/A	–	–	–
	35930	Ethiopia	N/A	–	–	–
	35998	Ethiopia	N/A	–	–	–
	36014	Ethiopia	N/A	–	–	–
	36029	Ethiopia	N/A	–	–	–
	25011	Nigeria	XIXd-1	2	1	4
	25698	Nigeria	N/A	–	–	–
	32929	Nigeria	XIXa-12	2	1	4
	40877	Nigeria	N/A	–	–	–
	13473	Jamaica	N/A	–	–	–
	13481	Jamaica	XIXa-8	2	4	4
	4739	Portugal	XIXa-9	1	2	4
	4750	Portugal	XIXa-9	2	2	4
Immunocompetent adults	42883	USA	XIXa-15	2	2	2
	43412	USA	N/A	–	–	–
	43415	USA	N/A	–	–	–
	44183	USA	N/A	–	–	–
	44675	USA	XIXa-17	2	1	2
	44878	USA	XIXa-16	2	2	2
	44884	USA	XIXc-1	2	3	3
	46903	USA	XIXa-13	2	3	4
	46904	USA	XIXa-10	3	2	4
	46905	USA	XIXa-18	2	1	2
	46906	USA	XIXd-1	1	1	4
	47250	USA	XIXa-7	2	1	4
Cats	9914	Peru	XIXa-1	2	2	4
	9948	Peru	XIXa-1	2	2	4
	45459	USA	XIXa-15	2	2	2
	46450	USA	XIXa-15	2	2	2
	7378	Slovakia	XIXa-11	2	2	4
	19391	Australia	XIXa-11	2	3	4
Calf	6544	Portugal	N/A	–	–	–
Rhesus macaque	34120	China	XIXa-12	3	1	4

N/A: PCR negative.

### PCR analysis of the gp60 gene

The recently developed nested PCR targeting the partial *C. felis gp60* gene was used in typing the *C. felis* isolates in the present study [10]. The primers used included GP60-Felis-F1 (5′-TTT CCG TTA TTG TTG CAG TTG CA-3′) and GP60-Felis-R1 (5′-ATC GGA ATC CCA CCA TCG AAC-3′) in primary PCR and GP60-Felis-F2 (5′-GGG CGT TCT GAA GGA TGT AA-3′) and GP60-Felis-R2 (5′-CGG TGG TCT CCT CAG TCT TC-3′) in secondary PCR. The amplicons were approximately 1,200 and 900 bp, respectively. The primary and secondary PCR mixtures contained 1 µl of DNA template (for primary PCR) or 2 µl of primary PCR product (for secondary PCR), 250 nM primary PCR primers or 500 nM secondary PCR primers, 2.5 mM MgCl_2_, 200 µM deoxynucleotide triphosphates, 1 × PCR buffer (15 mM Tris-HCl, 50 mM KCl and MgCl_2_; pH=8.0), and 1.5 U *Taq* polymerase in a total of 50 µl. To reduce PCR inhibition, 400 ng/µl of nonacetylated bovine serum albumin was used in the primary PCR. The amplification was performed on a GeneAmp PCR 9700 thermocycler (Applied Biosystems, Foster City, CA), consisting of an initial denaturation at 94°C for 5 min; 35 cycles at 94°C for 45 s, 52°C for 45 s, and 72°C for 80 s; and a final extension at 72°C for 10 min. Both positive (*C. felis* DNA from a human sample from Peru) and negative (molecular-grade water) controls were used in each PCR run. The secondary PCR products were visualized under the UV light after 1.5% agarose gel electrophoresis.

### Sequence analyses

All positive secondary PCR products were sequenced in both directions on an ABI 3130xl Genetic Analyzer (Applied Biosystems). The sequences were assembled using ChromasPro 2.1.6 (www.technelysium.com.au/ ChromasPro.html), manually cleaned and edited using BioEdit 7.0.5.3 (www.mbio.ncsu.edu/BioEdit/bioedit.html), and aligned with each other and reference sequences downloaded from GenBank using MUSCLE implemented in MEGA 10 (www.megasoftware.net/) with manual adjustments of sequence gaps. Simple tandem repeats presented in nucleotide sequences were identified using Tandem Repeats Finder 4.09 (http://tandem.bu.edu/trf/trf.html). To infer the phylogenetic relationship among subtypes of *C. felis*, a maximum-likelihood tree was constructed in MEGA 10 using the general time reversible model and gamma distribution in the calculation of substitution rates. The reliability of cluster formation was evaluated using the bootstrap method with 1,000 replicates. Unique nucleotide sequences derived from this study were deposited in GenBank under accession nos. MT458667–MT458684 and MT636067–MT636069.

## Results

### Amplification efficiency of the gp60 PCR

PCR products of the expected size were obtained from 44 (67%) of the 66 *C. felis* DNA preparations, including 37/58 from humans, 6/6 from cats, and 1/1 from a rhesus macaque. All 3 Indian and 5 Ethiopian isolates were negative in the *gp60* PCR. The success rates for *C. felis* in other countries were 21/27 for Peru, 11/14 for the United States, 3/3for Kenya, 2/4 for Nigeria, 2/3 for Portugal, 2/2 for China, 1/2 for Jamaica, 1/1 for Slovakia and 1/1 for Australia (Table 1). They generated PCR products of visibly different sizes as revealed by agarose gel electrophoresis.

### Sequence characteristics of the gp60 gene of C. felis

The multiple-sequence alignment generated showed the presence of 23 sequence types in four major groups (Table 2). Among the four groups, numerous nucleotide substitutions were present over the partial *gp60* gene. In addition to the differences in nucleotide differences, there were significant length differences in nucleotide sequences due mostly to the presence of repetitive sequences. Within the most variable group with 29 sequences, the presence of indels and single nucleotide substitutions (SNPs) led to the formation of 18 subtypes. Among them, the 6 subtypes from Peru differed from each other mostly in indels, while the 7 subtypes from the United States differed from each other in indels as well as SNPs. In contrast, the most conserved sequence group with 11 sequences had no nucleotide differences, whereas the other two minor sequence groups contained two sequences with only one SNP each. Significant nucleotide differences were found among the sequence groups.

**Table 2.** Differences in the number and nature of various tandem repeats among *Cryptosporidium felis* subtype families.

Subtype families	No. of sequences obtained	463–556 bp	667–679 bp	706–711 bp	770–910 bp	1015–1068 bp	1156–1167 bp
XIXa	29	1–3 copies of R1^a^	3 or 9-bp deletion	–	1–4 copies of R2^b^	–	2–5 GGT
XIXb	0	1 or 2 copies of R1	6 or 15-bp deletion	–	2–4 copies of R2	18-bp insertion	4 GGT
XIXc	2	2 copies of R1	15-bp deletion	–	3 copies of R2	–	3 GGT
XIXd	11	1 copy if R1	–	–	1 copy of R2	–	4 GGT
XIXe	2	1 copy of R1	–	6-bp insertion	1 copy of R2	36-bp deletion	4 GGT

^a^ 33-bp tandem repeat (CCACCTAGTGGCGGTAGTGGCGTGTCCCCTGCT) with an incomplete copy at the end.

^b^ 39-bp tandem repeat (AGCACAACTACGGCTACAG CGAGCACTGCGAGTTCGACA) with 0–5 single nucleotide substitutions.

The difference in the size of PCR products was due to the presence of sequence repeats in the *gp60* gene. A 33-bp repeat sequence 5′-CCA CCT AGT GGC GGT AGT GGC GTG TCC CCT GCT-3′ was found at the nucleotides ∼450–530 of the sequence alignment. The sequences generated had 1–3 copies of this repeat (the last copy was incomplete with nearly half of the length). Likewise, a 39-bp repeat sequence 5′-AGC ACA ACT ACG GCT ACA GCG AGC ACT GCG AGT TCG ACA-3′ was also observed at nucleotides ∼770–910 of the alignment. Sequences from the study had 1–4 copies of this repeat. These sequences also contained different copies of the trinucleotide repeat GTT at the 3′ end (Tables 1 and 2).

### Phylogenetic relationship among C. felis subtypes

**Figure 1.**
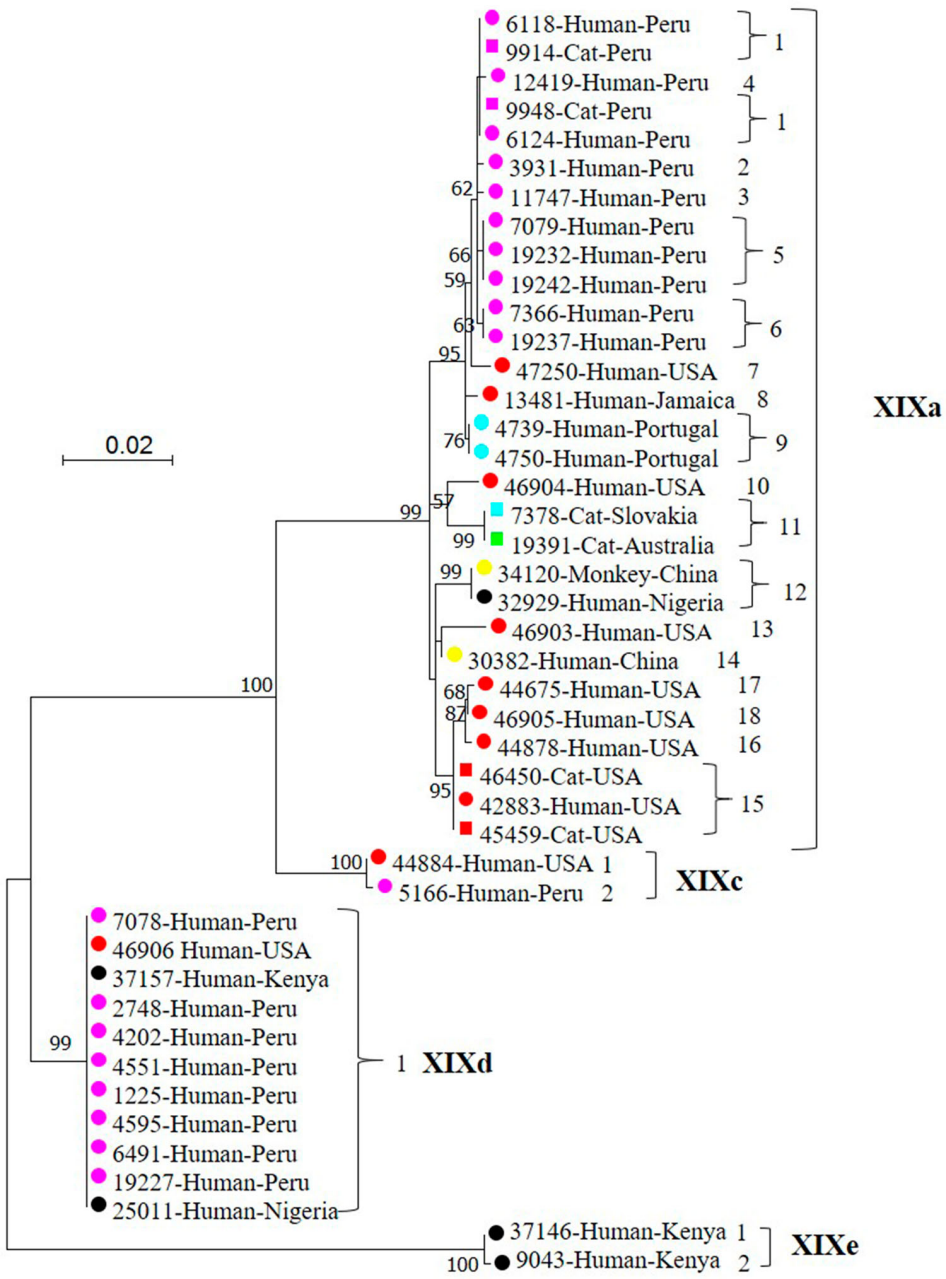
Phylogenetic relationship among four *Cryptosporidium felis* subtype families identified in the present study based on a maximum likelihood analysis of the partial *gp60* gene. Substitution rates were calculated by using the general time reversible model. Numbers on branches are percent bootstrapping values over 50 using 1,000 replicates. Round and square labels indicate samples from humans (including one from a monkey) and cats, respectively. Red, pink, blue, black, yellow and green labels indicate samples from North America, South America, Europe, Africa, Asia and Oceania, respectively.

In the maximum likelihood tree, the 44 DNA sequences formed four distinct clusters as expected, with 99–100% bootstrap support (Figure 1). Three of the four clusters were found in the previous study [10], while one cluster seen in the previous study, B, was absent from the present study. In accordance with the established nomenclature system for *Cryptosporidium* subtype families [11], the four clusters found in the present study were named as XIXa, XIXc, XIXd and XIXe (newly identified in the present study) subtype families, while the previous Cluster B was named as XIXb subtype family. In the present study, the dominant subtype families XIXa and XIXd contained 29 and 11 isolates, respectively, whereas the other two minor ones contained two isolates each. Among the four subtype families of *C. felis*, subtype families XIXd and XIXe were more distant from subtype families XIXa and XIXc (Figure 1). Within the subtype family XIXa, the 6 subtypes from Peru clustered together in the phylogenetic tree, while only 4 of the 7 subtypes from the United States formed a country-associated clade. In the former, two isolates from cats in Lima, Peru had the same subtype (XIXa-1) as two isolates from HIV+ patients in the same city (Figure 1).

### Distribution of C. felis subtypes by host

Sequences obtained from all 6 feline isolates clustered within the subtype family XIXa, while human-derived isolates could be found in all four clusters. Among the 25 *C. felis* isolates from humans in Lima, Peru, there were no obvious differences in subtype distribution between children and HIV+ adults. The XIXa subtype family contained one nonhuman primate isolate from China, which generated a sequence identical to the one from a human in Nigeria. With the exception of a calf sample, all PCR failures (21 in 66) occurred with human isolates (Table 1).

### Distribution among C. felis subtypes by country

**Figure 2.**
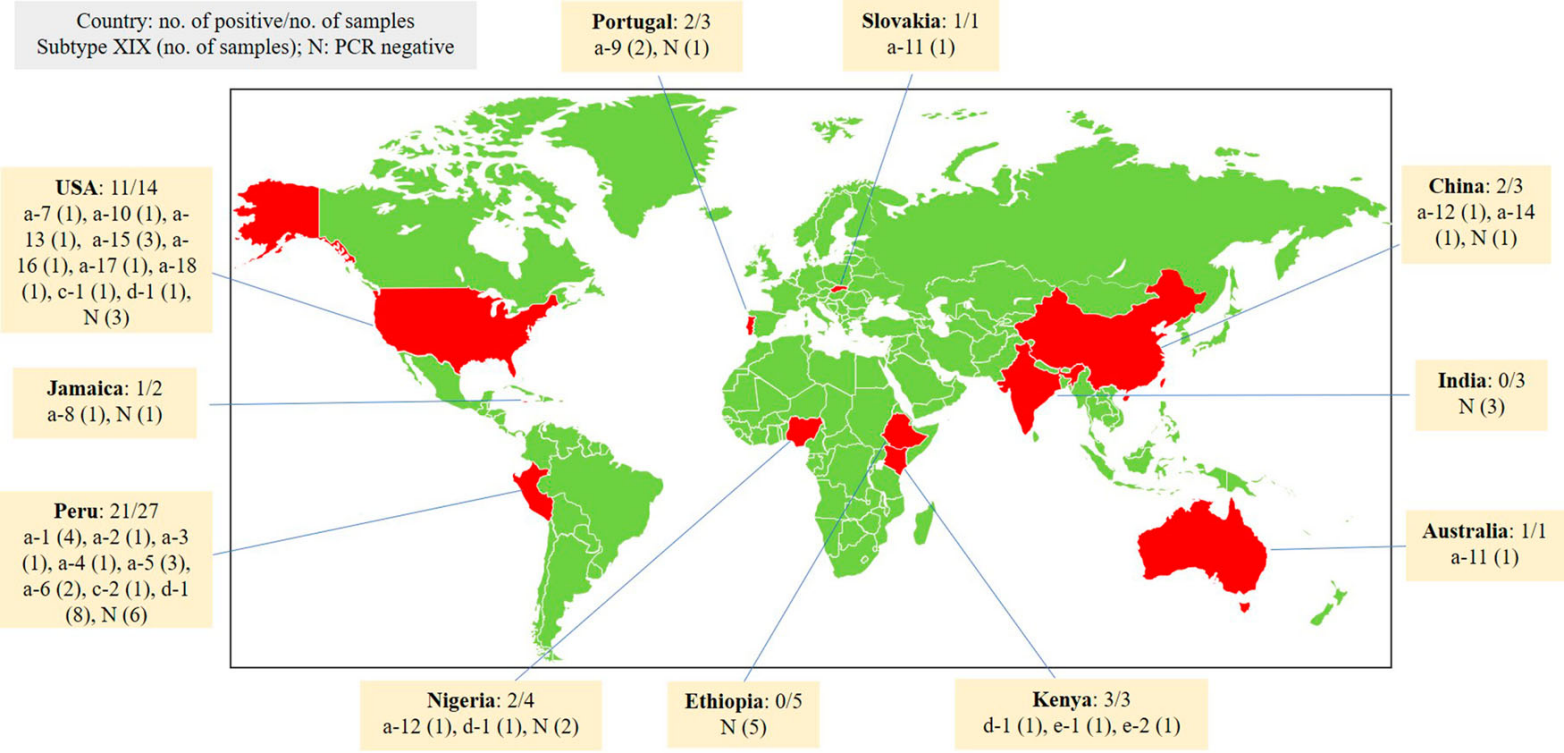
Geographic distribution of *Cryptosporidium felis* subtypes detected in this study.

Among the four subtype families generated from this study, XIXa was found in the United States (9), Jamaica (1), Peru (12), Portugal (2), Slovakia (1), China (1), and Australia (1); XIXc was found in the United States (1) and Peru (1); XIXd was found in the United States (1), Peru (8), Kenya (1), and Nigeria (1); and XIXe was only found in Kenya (2) (Table 1, Figure 2).

Among the 18 *C. felis* XIXa subtypes, XIXa-1 (4), XIXa-2 (1), XIXa-3 (1), XIXa-4 (1), XIXa-5 (3), and XIXa-6 (1) were found in Peru; XIXa-7 (1), XIXa-10 (1), XIXa-13 (1), XIXa-15 (2), XIXa-16 (1), XIXa-17 (1), and XIXa-18 (1) in the United States; XIXa-8 (1) in Jamaica; XIXa-9 (2) in Portugal; XIXa-11 (1) in Slovakia; XIXa-12 (1) in Nigeria; XIXa-12 (1) and XIXa-14 (1) in China; and XIXa-11 (1) in Australia (Table 1, Figure 2).

## Discussion

Studies on the transmission of *C. felis* in humans have been hampered for a long time by the lack of subtyping tools until the recent development of a *gp60*-based PCR for this zoonotic species [10,11,18]. In this study, the newly developed subtyping tool was utilized in genetic characterization of diverse isolates from several hosts and countries and a nomenclature system was developed in naming the subtype families of *C. felis* identified thus far. Results of this analysis show a high genetic diversity of this parasite and, together with the data from the published study, the possible occurrence of human-adapted populations of *C. felis*.

**Figure 3.**
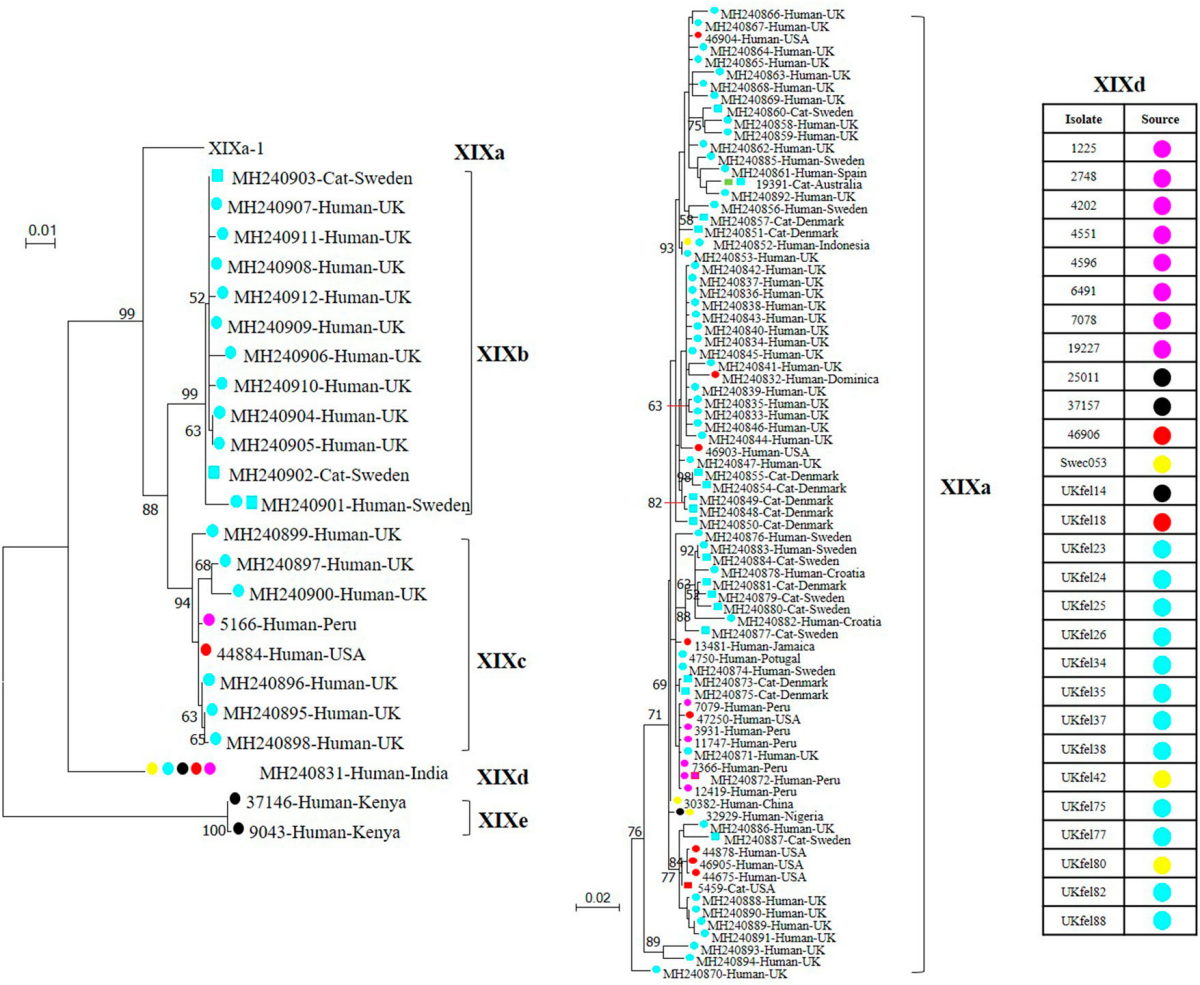
Phylogenetic relationship of known *Cryptosporidium felis* subtypes based on a maximum likelihood analysis of the partial *gp60* gene. Substitution rates were calculated using the general time reversible model. Numbers on branches are percent bootstrap values over 50 from analysis with 1000 replicates. Round and square labels indicate samples from humans (including one from a monkey) and cats, respectively. Red, pink, blue, black, yellow and green labels indicate samples from North America, South America, Europe, Africa, Asia and Oceania, respectively. Phylogenetic relationships of five subtype families and the XIXa subtypes are shown in the left tree and the right tree, respectively. The sources of subtype XIXd-1 isolates are indicated in the right table.

The *gp60* gene of *C. felis* appears to be highly polymorphic compared to its orthologs in other *Cryptosporidium* spp. This is reflected by the existence of four subtype families among the small number of samples characterized in the present study, and the extensive sequence differences among subtype families and subtypes in both SNPs and indels. This finding is in agreement with recent subtyping data generated by *gp60* sequence analysis of 128 *C. felis* isolates [10]. Subtyping data from the two studies have shown that *C. felis* consists of at least five subtype families in two large clades in a maximum-likelihood tree (Figure 3). The first clade consists of subtype families XIXa, XIXb, and XIXc with extensive sequence variations within each subtype family, while the second one consists of XIXd and XIXe with relatively conserved sequences.

Although *C. felis* is considered a feline-specific parasite, it has been reported in significant numbers of humans (both immunocompromised and immunocompetent persons) and a few nonhuman primates, calves, horses, foxes [19–24]. Data generated in this study suggest the immune status of patients is irrelevant to the human infections with *C. felis* subtypes. This is supported by finding subtype families XIXa, XIXc and XIXd in both immunocompromised and immunocompetent patients, including reasonable number of isolates examined from children and AIDS patients in Lima, Peru (Table 1). As all *gp60* PCR failures occurred with non-feline isolates in this study, some of these hosts could have only light infections of *C. felis*. The identification of *C. felis* in faecal specimens was based on nested PCR analysis of the SSU rRNA gene, which has five copies per genome, compared with one copy of the *gp60* gene.

Data generated from this study suggest that there could be host adaptation within *C. felis*. This is supported by the observation of all 11 investigated subtype family XIXd isolates were derived from humans, whereas both human and feline subtypes could be observed in subtype families XIXa and XIXb. Hence, *C. felis* subtype family XIXd might be adapted to humans. This finding is supported by the published data; in which all 28 XIXd isolates were from humans, while the subtype families XIXa and XIXb contained isolates from both humans and cats (Table 3, Figure 3) [10]. Notably, no feline isolates were found in the XIXc, XIXd and XIXe subtype families, but only a small number of isolates were characterized for each of them, especially XIXd and XIXe. Therefore, further epidemiological and experimental data are needed to assess the prevalence and infectivity of the subtype families XIXc, XIXd and XIXe in felines. Host adaption is common in some zoonotic *Cryptosporidium* spp. at the *gp60* subtype family level [25], such as *C. parvum* subtype family IIc in humans, *C. hominis* subtype family Ik in equine animals, and *C. ubiquitum* subtype family XIIa in small ruminants [8,13].

**Table 3.** Distribution of *Cryptosporidium felis* subtypes and copy numbers of major tandem repeats in published studies.^a^

Subtype^b^	GenBank accession no.	Host (no. of isolates)	Country (no. of isolates)	No. of 33-bp repeat.	No. of 39-bp repeat	No. of GGT
XIXa-1	MH240872	Human (3) / Cat (2)	Peru (5)	2	2	4
XIXa-2	MT458673	Human (1)	Peru (1)	2	2	4
XIXa-3	MT636067	Human (1)	Peru (1)	2	2	4
XIXa-4	MT458672	Human (1)	Peru (1)	2	2	4
XIXa-5	MT458669	Human (3)	Peru (3)	2	3	4
XIXa-6	MT458674	Human (1)	Peru (1)	2	3	4
XIXa-7	MT458676	Human (1)	USA (1)	2	1	4
XIXa-8	MT458670	Human (1)	Jamaica (1)	2	4	4
XIXa-9	MT458671	Human (2)	Portugal (2)	2 or 3	2	4
XIXa-10	MT458681	Human (1)	USA (1)	3	2	4
XIXa-11	MT458675	Cat (2)	Slovakia (1) / Australia (1)	2	2	4
XIXa-12	MT458684	Human (1) / Monkey (1)	Nigeria (1) / China (1)	2 or 3	1	4
XIXa-13	MT458680	Human (1)	USA (1)	2	3	4
XIXa-14	MT458682	Human (1)	China (1)	2	2	2
XIXa-15	MT458679	Human (3)	USA (3)	2	2	2
XIXa-16	MT458678	Human (1)	USA (1)	2	2	2
XIXa-17	MT458677	Human (1)	USA (1)	1	2	2
XIXa-18	MT636068	Human (1)	USA (1)	2	2	4
XIXa-19	MH240832	Human (1)	Dominican Republic (1)	1	3	3
XIXa-20	MH240833	Human (1)	UK (1)	1	2	3
XIXa-21	MH240834	Human (1)	UK (1)	1	2	3
XIXa-22	MH240835	Human (1)	UK (1)	2	3	3
XIXa-23	MH240836	Human (2)	UK (2)	2	3	3
XIXa-24	MH240837	Human (1)	UK (1)	1	3	3
XIXa-25	MH240838	Human (1)	UK (1)	3	3	3
XIXa-26	MH240839	Human (1)	UK (1)	3	2	3
XIXa-27	MH240840	Human (1)	UK (1)	1	2	3
XIXa-28	MH240841	Human (1)	UK (1)	2	2	3
XIXa-29	MH240842	Human (1)	UK (1)	2	2	3
XIXa-30	MH240843	Human (1)	UK (1)	2	2	3
XIXa-31	MH240844	Human (1)	UK (1)	0	2	3
XIXa-32	MH240845	Human (1)	UK (1)	2	3	3
XIXa-33	MH240846	Human (1)	UK (1)	2	3	3
XIXa-34	MH240847	Human (1)	UK (1)	2	2	4
XIXa-35	MH240848	Cat (1)	Denmark (1)	1	2	4
XIXa-36	MH240849	Cat (1)	Denmark (1)	2	2	4
XIXa-37	MH240850	Cat (3)	Denmark (3)	2	3	4
XIXa-38	MH240851	Cat (1)	Denmark (1)	2	2	4
XIXa-39	MH240852	Human (2)	Indonesia (1) / UK (1)	2	2	4
XIXa-40	MH240853	Human (1)	UK (1)	3	2	4
XIXa-41	MH240854	Cat (1)	Denmark (1)	2	2	4
XIXa-42	MH240855	Cat (1)	Denmark (1)	3	1	4
XIXa-43	MH240856	Human (1)	Sweden (1)	2	2	4
XIXa-44	MH240857	Cat (1)	Denmark (1)	2	2	4
XIXa-45	MH240858	Human (1)	UK (1)	2	3	4
XIXa-46	MH240859	Human (1)	UK (1)	1	1	4
XIXa-47	MH240860	Cat (1)	Sweden (1)	1	1	4
XIXa-48	MH240861	Human (1)	Spain (1)	2	3	4
XIXa-49	MH240862	Human (1)	UK (1)	2	3	4
XIXa-50	MH240863	Human (1)	UK (1)	2	3	4
XIXa-51	MH240864	Human (1)	UK (1)	1	3	4
XIXa-52	MH240865	Human (1)	UK (1)	2	3	4
XIXa-53	MH240866	Human (1)	UK (1)	1	1	4
XIXa-54	MH240867	Human (1)	UK (1)	2	1	4
XIXa-55	MH240868	Human (1)	UK (1)	2	1	4
XIXa-56	MH240869	Human (1)	UK (1)	1	2	4
XIXa-57	MH240871	Human (1)	UK (1)	2	3	4
XIXa-58	MH240873	Cat (1)	Denmark (1)	2	2	4
XIXa-59	MH240874	Human (1)	Sweden (1)	2	2	4
XIXa-60	MH240875	Cat (1)	Denmark (1)	1	2	4
XIXa-61	MH240876	Human (1)	Sweden (1)	2	1	4
XIXa-62	MH240877	Human (1)	Sweden (1)	2	2	4
XIXa-63	MH240878	Human (1)	Croatia (1)	2	2	4
XIXa-64	MH240879	Cat (2)	Sweden (2)	2	3	4
XIXa-65	MH240880	Human (1)	Sweden (1)	1	3	4
XIXa-66	MH240881	Human (1)	Denmark (1)	2	2	4
XIXa-67	MH240882	Human (1)	Croatia (1)	2	3	4
XIXa-68	MH240883	Human (1)	Sweden (1)	2	3	4
XIXa-69	MH240884	Cat (1)	Sweden (1)	2	3	4
XIXa-70	MH240885	Human (1)	Sweden (1)	2	3	4
XIXa-71	MH240886	Human (1)	UK (1)	2	3	4
XIXa-72	MH240888	Human (1)	UK (1)	2	1	4
XIXa-73	MH240889	Human (1)	UK (1)	2	3	3
XIXa-74	MH240890	Human (1)	UK (1)	1	2	4
XIXa-75	MH240891	Human (1)	UK (1)	0	3	4
XIXa-76	MH240892	Human (1)	UK (1)	1	2	4
XIXa-77	MH240870	Human (1)	UK (1)	3	4	4
XIXa-78	MH240887	Cat (1)	Sweden (1)	2	1	4
XIXa-79	MH240893	Human (1)	UK (1)	3	4	5
XIXa-80	MH240894	Human (1)	UK (1)	2	1	4
XIXb-1	MH240901	Human (1) / Cat (1)	Sweden (2)	2	3	4
XIXb-2	MH240902	Cat (1)	Sweden (1)	1	2	4
XIXb-3	MH240903	Cat (1)	Sweden (1)	2	2	4
XIXb-4	MH240904	Human (1)	UK (1)	2	2	4
XIXb-5	MH240905	Human (1)	UK (1)	2	3	4
XIXb-6	MH240906	Human (1)	UK (1)	2	2	4
XIXb-7	MH240907	Human (1)	UK (1)	2	2	4
XIXb-8	MH240908	Human (1)	UK (1)	2	2	4
XIXb-9	MH240909	Human (1)	UK (1)	2	2	4
XIXb-10	MH240910	Human (1)	UK (1)	2	2	4
XIXb-11	MH240911	Human (1)	UK (1)	2	4	4
XIXb-12	MH240912	Human (1)	UK (1)	2	2	4
XIXc-1	MT458667	Human (1)	USA (1)	2	3	3
XIXc-2	MT458668	Human (1)	Peru (1)	2	3	3
XIXc-3	MH240895	Human (1)	UK (1)	2	3	3
XIXc-4	MH240896	Human (1)	UK (1)	2	3	3
XIXc-5	MH240897	Human (1)	UK (1)	2	2	3
XIXc-6	MH240898	Human (1)	UK (1)	1	3	3
XIXc-7	MH240899	Human (1)	UK (1)	2	3	3
XIXc-8	MH240900	Human (1)	UK (1)	2	1	3
XIXd-1	MH240831	Human (28)	World-wide^a,c^ (28)	1	1	4
XIXe-1	MT636069	Human (1)	Kenya (1)	1	1	4
XIXe-2	MT458683	Human (1)	Kenya (1)	1	1	4

^a^ Including data from the present study and [10].

^b^ Subtypes XIXa-1, XIXa-19–XIXa-80, XIXb-1–XIXb-12 and XIXc-3–XIXc-8 were detected from the previous study.

^c^ Including USA (2), Peru (8), UK (11), Sudan (1), Kenya (1), Nigeria (1), India (3) and Pakistan (1).

Potential geographic segregation exists in the distribution of some *C. felis* subtypes. This is supported by the presence of genetically related clusters of *C. felis* subtypes in the same geographic area. For example, subtypes XIXa-1–XIXa-6 were only detected in Peru, and XIXa-13–XIXa-18 were only found in the United States, whereas subtypes XIXa-8, XIXa-9 and XIXe were found in Jamaica, Portugal and Kenya, respectively. Similarly, subtypes in the XIXb subtype family have been only detected in Europe (the United Kingdom and Sweden) thus far (Figure 3) [10]. Moreover, due likely to the presence of geographically unique subtypes, all five Indian and three Ethiopian *C. felis* isolates were PCR negative. However, data generated in this and previous studies also indicate that *C. felis* subtype XIXd-1 has a world-wide distribution, as it has been detected in geographically distant areas, such as the United States, United Kingdom, Peru and several African and Asian countries (Figure 1) [10]. Nevertheless, these suggestions are based on the characterization of a small number of samples from limited areas. More data are needed to substantiate the presence of geographic differences in the distribution of *C. felis* subtypes.

In conclusion, 44 *C. felis* isolates were successfully characterized using the newly developed *gp60* subtyping tool and a nomenclature system was developed for naming *C. felis* subtypes. The data generated from the present and published studies both suggest potential host-adaptation and geographic isolation within *C. felis* at the subtype level. Further studies involving more samples from diverse areas, especially those from cats and other animals, are needed to confirm these observations and to improve our understanding of the transmission of this important zoonotic pathogen.
